# Effects of Primary Blast Overpressure on Retina and Optic Tract in Rats

**DOI:** 10.3389/fneur.2016.00059

**Published:** 2016-04-25

**Authors:** James DeMar, Keith Sharrow, Miya Hill, Jonathan Berman, Thomas Oliver, Joseph Long

**Affiliations:** ^1^Blast Induced Neurotrauma Branch, Center for Military Psychiatry and Neuroscience, Walter Reed Army Institute of Research, Silver Spring, MD, USA; ^2^Medical Countermeasures Systems, Ft. Detrick, Frederick, MD, USA; ^3^Clinical Pharmacology Department, Walter Reed Army Institute of Research, Silver Spring, MD, USA; ^4^Clinical Research Unit, Uniformed Services University of the Health Science (USUHS), Bethesda, MD, USA

**Keywords:** blast, neurotrauma, retina, optic tract, rat

## Abstract

Blast has been the leading cause of injury, particularly traumatic brain injury and visual system injury, in combat operations in Iraq and Afghanistan. We determined the effect of shock tube-generated primary blast on retinal electrophysiology and on retinal and brain optic tract histopathology in a rat model. The amplitude of a- and b-waves on the electroretinogram (ERG) for both right and left eyes were measured prior to a battlefield simulation Friedlander-type blast wave and on 1, 7, and 14 days thereafter. Histopathologic findings of the right and left retina and the right and left optic tracts (2.8 mm postoptic chiasm) were evaluated 14 days after the blast. For two experiments in which the right eye was oriented to the blast, the amplitude of ERG a- and b-waves at 7 days post blast on the right side but not on the left side was diminished compared to that of sham animals (*P* = 0.005–0.01) Histopathologic injury scores at 14 days post blast for the right retina but not the left retina were higher than for sham animals (*P* = 0.01), and histopathologic injury scores at 14 days for both optic tracts were markedly higher than for shams (*P* < 0.0001). Exposure of one eye to a blast wave, comparable to that causing human injury, produced injury to the retina as determined by ERG and histopathology, and to both postchiasmatic optic tracts as determined by histopathology. This model may be useful for analyzing the effect of therapeutic interventions on retinal damage due to primary blast waves.

## Introduction

Blast has been the leading cause of injury in the conflicts in Iraq and Afghanistan (1). From 2000 through the end of 2014, more than 320,000 Service-members have suffered at least one traumatic brain injury (TBI) (2) many of which have been due to exposure to an explosive device (3). Blast-related injuries can be categorized as primary, secondary, and tertiary with respect to the blast wave. A primary injury results from the blast wave itself and subsequent changes in air pressure. The intense over-pressurization wave applies force to tissues that results in rapid deformation and disruption (3). Secondary injury is due to other objects that are set in motion by the blast then impacting the body; tertiary injury is, conversely, the body being set in motion by the blast and striking other objects (1). Visual impairment is a common symptom of blast-injured military veterans. In a retrospective record, review of 50 blast-injured patients admitted to the Palo Alto Polytrauma Rehabilitation Center, more than half reported each of vision complaints, light sensitivity, and reading deficits, and more than half were found to have saccades or abnormal accommodation (1). In a prospective case series of 46 blast-exposed veterans, 20 (43%) had closed/non-penetrating ocular injury (4).

Specifying the effect of the blast wave on any part of the visual system is complicated by the complex nature of both cause and effect. Primary, secondary, or tertiary blast insults could occur together. A part of the visual system could be directly injured or could degenerate secondary to distant injury via anterograde (5, 6) or retrograde (7, 8) mechanisms.

Despite the lifelong disability that vision losses represent, there are only a modest number of studies in animals that have attempted to assess the effect of blast waves on the visual system (see Table 1 and its summary in Discussion).

**Table 1:** **Literature review of visual system injury by simulated blast**.

Reference	Animal	Injury-method^a^	Outcome measures and time recorded post injury	Neuronal degeneration
Petras et al. (28)	Rat	Single blast wave by shock tube, right side on. 12–22 psi	Brain histopathology. Vision-based behavioral tests. 14 days	YES, for brain visual centers
Koliatsos et al. (29)	Mouse	Single blast wave by shock tube, supine face on. 27 psi	Retina and brain histopathology. Vision-based behavioral tests. 5–14 days	YES, for retina and brain visual centers
Hines-Beard et al. (23)	Mouse	Air-blast directly to left eye. 23–30 psi	Eye gross pathology. Intraocular pressures. Optical coherence tomography. Visual acuity (optokinetics). 3–28 days	YES, for retina and optic nerve
Jiang et al. (12)	Mouse	Air-blast directly to left eye. Postinjury treatment with a β-adrenergic agonist. 23 psi	Retina histopathology and immunohistochemistry. Apoptotic protein ELISAs. 4-72 h	YES, for retina
Mohan et al. (24)	Mouse	Air-blast inside an open chamber, left side on. 20 psi	Retina histopathology and optic nerve electron microscopy. Pupil constriction response. Tear production. ERGs. Optical coherence tomography. Apoptotic protein ELISAs. Oxidative stress marker assays. 1–24 h. 7 days. 4–10 months	YES, for retina and optic nerve
Zou et al. (11)	Rat	Single blast wave by explosive charge detonations, prone face on. 26–70 psi	Retina histopathology and immunohistochemistry. Cytokine immunoassay arrays. Apoptotic and edema protein Westerns. Neurotransmitter assays. 1–14 days	YES, for retina
Bricker-Anthony et al. (20)	Mouse	Air-blast directly to left eye. 26 psi	Retina and optic nerve histopathology and immunohistochemistry. ERGs. Optical coherence tomography. Oxidative stress marker assays. 3–28 days	YES, for retina and optic nerve
Bricker-Anthony et al. (21)	Mouse	Air-blast directly to left eye. 23–30 psi	Retina and optic nerve histopathology and immunohistochemistry. ERGs. Visual acuity (optokinetics). Optical coherence tomography. Oxidative stress marker assays. 3–28 days	YES, for retina and optic nerve
Wang et al. (30)	Rat	Single blast wave by shock tube, right side on. 17 psi	Retina and optic nerve histopathology and immunohistochemistry. 3–48 h	YES, for retina and optic nerve
Dutca et al. (13)	Mouse	Air-blast inside an open chamber, left side on. Postinjury treatment with a nicotinamide phosphoribosyltransferase agonist. 20 psi	Retina histopathology and dendritic field analysis. ERGs. Single retinal cell/multi-electrode array recordings. Intraocular pressures. Optical coherence tomography. 1–16 weeks	YES, for retina
Jiang et al. (31)	Mouse	Air-blast directly to left eye. Postinjury treatment with a β-adrenergic agonist. 26 psi	Retina growth factor and apoptotic protein and cytokine ELISAs and Western blots. 4–72 h	YES, for retina
Reiner et al. (14)	Mouse	Air-blast directly to skull’s parietal – squamosal area. Postinjury treatment with a cannabinoid receptor agonist. 50–60 psi	Retina and brain histopathology and immunohistochemistry. Visual acuity (optokinetics). 3 days–11 weeks	YES, for retina and brain visual centers
Choi et al. (32)	Rat	Single or repetitive blast waves by shock tube, right side on. 10 psi	Retina and optic nerve immunohistochemistry. 4 days	YES, for retina and optic nerve
Bricker-Anthony et al. (22)	Mouse	Air-blast directly to left eye, but only contralateral (right eye) tested. 26 psi	Eye gross pathology. Retina and optic nerve histopathology and immunohistochemistry. ERGs. Visual acuity (optokinetics). Optical coherence tomography. 3–28 days	YES, just for retina
Guley et al. (33)	Mouse	Air-blast directly to skull’s parietal – squamosal area. 20–60 psi	Retina, optic nerve, and brain histopathology and immunohistochemistry. Visual acuity (optokinetics). 3 days–11 weeks	YES, for retina, optic nerve, and brain visual centers

*^a^psi, pounds per square inch*.

There are two major experimentally sound models of blast: open-field explosive blasts and shock tubes (9, 10). An open-field blast occurs when an explosive device is detonated in an open area. Although an open field blast is the most accurate representation of the complex clinical situation, the shock waves produced are complex, and it is difficult to evaluate primary blast effects by themselves; and only one animal study has adopted this approach for eye injury studies (11). Shock tubes using compressed gas are a widely used alternative to explosive blasts because shock tubes are safer and easier to control. These tubes consist of a “driver” section at the closed end, separated from a “driven” section by a frangible or breakable diaphragm composed of mylar or cellulose acetate. The process begins with pressurization of the closed off driver section. When the pressure reaches a critical level, the diaphragm ruptures creating a shockwave of known intensity.

Our principal interest is evaluating the effect of the primary blast wave on the initial neurons in the afferent visual system: photoreceptor and associated retinal cells and the brain optic tract. Our ultimate interest is to assess the ability of drugs to ameliorate blast-induced retinal degeneration, a field with to our knowledge only three present reports (12–14). To this end, we have devised a shock tube utilizing compressed air from which only the primary blast wave and not secondary and tertiary forces are generated, and which generates a blast wave with Friedlander wave form and thus is comparable to that experienced in combat (15). Since we intend to use this model to evaluate drug treatment of retinal and optic tract damage, rats, not mice were exposed to the blast as drug pharmacokinetics are more reliably performed on these larger animals with slower metabolism rates. We report below the effect of two experimental shock-tube blast models – one side of the face exposed to the blast and the front of the face exposed to the blast – on retinal electrophysiology as well as retinal and brain optic tract histopathology in rats.

## Materials and Methods

### Induction of Eye and Brain Injuries Using Exposure to Blast Waves

**Figure 1:**
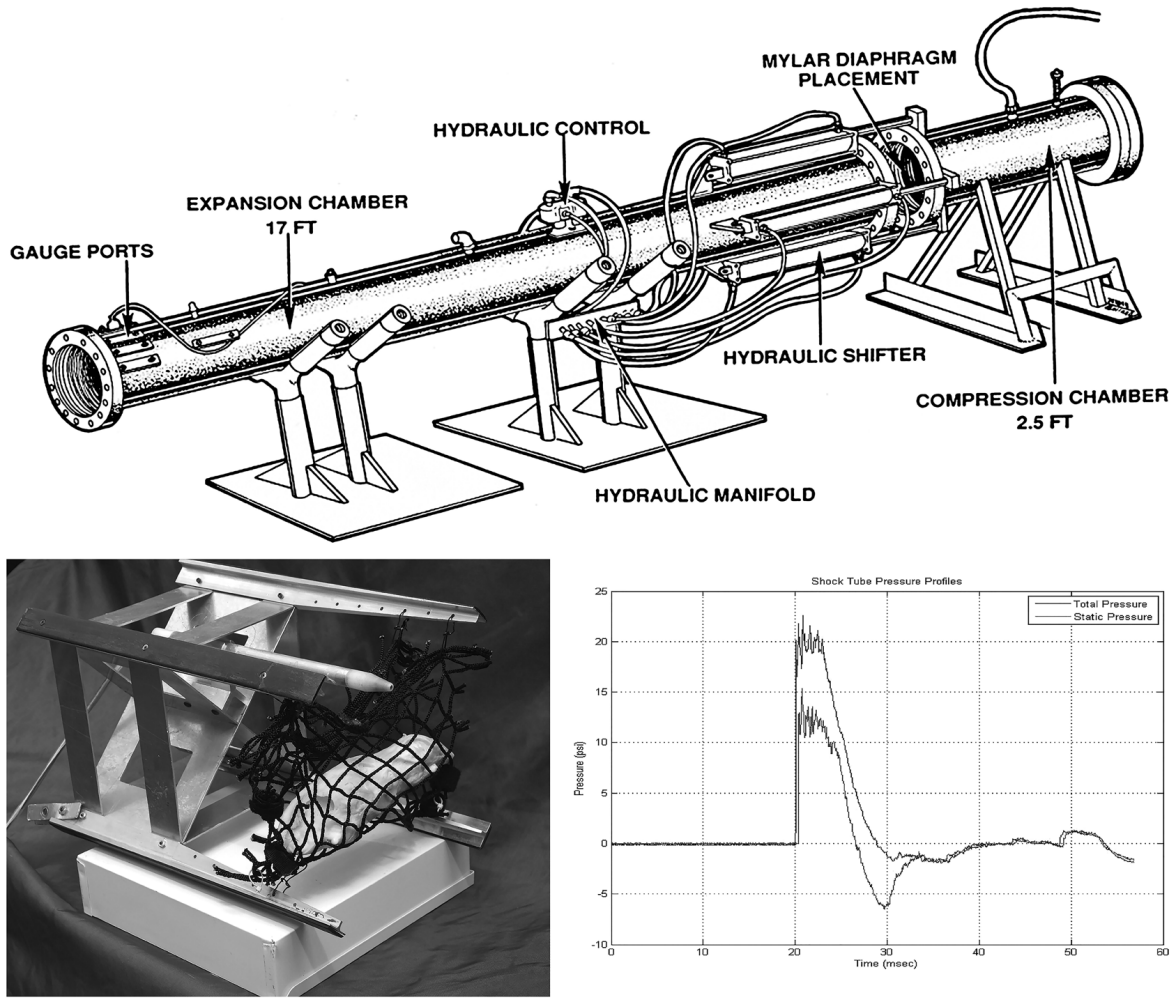
**Schematic diagram of the compressed air-driven shock tube for generation of blast wave injuries to the eyes and brains of rats**. Upper: diagram of the compressed air-driven shock tube. Bottom left: the animal holder consists of a metal sled equipped with a nylon mesh sling (mock rat is displayed inside as mounted in a right blast position) that is inserted down into expansion chamber before blast wave exposure. Bottom right: during blasting, the shock tube delivers a static pressure of 20 psi at the position of the animal inside the expansion chamber. The blast wave travels by the rat with a 6 ms duration.

Adult male Sprague-Dawley rats (2 months old: Charles River Laboratories, Inc.) were placed under brief anesthesia using isoflurane gas. Anesthetized animals were put in a prone transverse position inside a nylon mesh sling that is secured to a metal frame sled (Figure 1, bottom left). For experiments in which the whole face is exposed to the blast (“face blast”/“face-to-blast” experiments), rats were positioned with the face aligned with the long axis of the sled such that right and left eyes are expected to incur equal injuries. For experiments in which one side of the face (the right side) was exposed to the blast (“right blast”/“right-to-blast” experiments), rats were positioned with the right side of the body perpendicular to the long axis of the sled such that the right eye faced the oncoming blast wave. For right-blast experiments, the left eye, which was anticipated to incur less severe injuries or none, served as a contralateral control.

Blast exposure was carried out using a compressed air-driven shock tube as described previously (16). Briefly, after 4% isoflurane anesthesia in an induction box for up to 8 min (O_2_ flow rate 1.5 L/min), rats were immediately tautly secured in a prone position in coarse mesh netting 2.5 ft within the mouth of the 15 ft long and 1 ft internal diameter expansion chamber (Figure 1, upper) either facing the driver section (“face-to-blast”) or with the right side of the head/body facing the driver section (“right-to-blast”). While under isoflurane anesthesia, there is a reduced tonic contraction of the orbicularis oculi muscles and the rat’s eyelids tend to remain wide open; and thus the surface of their eyes receives the brunt of the blast wave. Rats were exposed to single shockwave. Blast overpressure flow conditions were recorded using piezoresistive pressure transducers (Meggit Inc., San Juan Capistrano, CA, USA) mounted in the rat holder, which provided measurements of total and side-on pressure waveforms. In these studies, the Friedlander-type blast wave typically had a 6 ms duration and a peak static pressure of 20 psi (Figure 1, bottom right). Blasted rats were immediately removed from the shock tube and monitored on a thermal blanket during recovery. Animals exhibiting stable respiration and awakening signs were returned to their housing cages. Sham animals were subjected to isoflurane anesthesia and recovery procedures as described above, but not to blast waves. Blasted and sham rats were then used for electroretinogram (ERG) or retinal and optic tract histopathology, as described below.

### Electroretinogram Recordings of Sham and Blasted Rats

Rats were adapted in full darkness for at least 5 h, prior to being ERG tested. The dark adaptation was done to prime the retina light signaling responses and reduce retinal neuron background noise. Preliminary experiments with 5 h vs. 16 h of dark adaptation (*n* = 6) showed only small (<15%) decreases in the amplitudes and increases in the variability of ERG recordings compared to the more convenient 5-h stabilization period. Rats were then placed under anesthesia using isoflurane gas and pupils dilated using drops of 0.5% tropicamide and 2.5% phenylephrine (cholinergic antagonist and α-adrenergic agonist, respectively). The rats’ eyes were also numbed with drops of 0.5% propracaine. The animal, while maintained on gas anesthesia through a nose cone, was placed on a thermal blanket and a ground electrode fixed to the tail and reference electrodes to both cheeks, using short sub-dermal pins. Recording electrodes were attached to each cornea by placing the fine silver wire leads under contact lens affixed with 2% methylcellulose solution. The rat was laid prone with its face fully inserted into the light stimulus dome of a Handheld Multispecies electroretinogram (ERG) unit (HMs-ERG; Ocuscience, Inc.). This device was specifically modified for doing rat ERGs by the manufacturer, which provided us with onsite training, quality control (QC) data, and utilization citations from more than a dozen established vision research labs. The eyes were then given a scotopic full field flash ERG exam (i.e., dark adapted response), using a light stimulus procedure that exposes the eyes to a series of white light flashes of six increasing intensities (0.1, 0.3, 1, 3, 10, and 25 cd.s/m^2^), with each repeated one to four times (and the results averaged) at an interval of 10 s and a duration of 5 ms, and having a ramp spacing of 30–60 s. This procedure was recommended by the manufacturer for obtaining reliable ERG results on rats (i.e., a broad-range flash response curve). ERG responses arising from each eye were recorded simultaneously by computer and the peak voltage amplitudes of the underlying a- and b-wave forms are derived to judge the functional status of the retina photoreceptors and bipolar/amacrine neurons, respectively. After the ERG exam, an ophthalmological ointment (artificial tears) was applied to each eye to alleviate corneal irritation; then to protect their dilated eyes from bright light damage, the rats were kept in darkness for at least several hours until they were recovered from anesthesia and pupil constriction reflex was restored. Animals were thereafter returned to their normal housing cages under standard lighting conditions.

Rats were given an ERG exam at 1 day prior to blast wave exposure to establish their baseline light stimulus responses, and then retested once at 1, 7, and 14 days post blast. Peak amplitudes for a- and b-wave responses only at the light flash intensity of 3 cd s/m^2^ were used to evaluate overall ERG data with time. This flash intensity is recommended by the International Society for Clinical Electrophysiology of Vision (ISCEV) as an optimal light stimulus for doing ERG recordings in research animals and humans (17).

### Histopathology of Eyes and Brains from Shams and Blasted Rats

At 14 days post blast wave exposure, after a final ERG exam, rats were euthanized for tissue collection. Animals were anesthetized with isoflurane and then perfused transcardially with saline, resulting in euthanasia by blood exsanguination, followed by phosphate buffered 4% paraformaldehyde 0.15% (wt/vol) picric acid. After perfusion, eyes and whole brain were removed. Tissues were then subjected to further processing over several days with other fixative reagents. To toughen the globes, eyes were postfixed for 6 h in 2% trichloroacetic acid, 2% zinc chloride, and 20% isopropanol as made up in 4% paraformaldehyde. Postfixed eyes were washed with phosphate buffered saline followed by 50% ethanol and then stored in 70% ethanol. Eyes were cut in a single horizontal section (5 μm) through the pupil’s central axis in line with the head of the optic nerve. Brains were cryoprotected with a 20% sucrose solution in Phosphate buffer and then cut in 11 evenly spaced coronal sections (30 μm) through the cerebrum ending at the back of the midbrain, to cover all underlying visual processing centers. Fixed sections were made into slides and stained with hematoxylin and eosin (eyes) and silver (brains: optic tract, dorsal lateral geniculate body, superior colliculus, and visual cortex). Prepared slides were examined under an axial light microscope equipped with an image capture camera and a computer having image processing software. For eyes, distinct neuronal layers making up the retina (e.g., ganglion, bipolar/amacrine, and photoreceptor cells) were examined. Eyes were evaluated for retinal degeneration and all non-neuronal components ignored (e.g., cornea, iris, and lens). The approximate anatomic location of brain visual structures (optic tract, geniculate nucleus, superior colliculus, and visual cortex) was in lateral distance from the Bregma and midline sutures – and depth from the dura (i.e., surface of skull): optic tract −3.24 mm Bregma; 3.9 mm × 7.8 mm; dorso lateral geniculate nucleus −4.68 mm Bregma; 3.6 mm × 5.0 mm; superior colliculus −6.48 mm Bregma; 1.4 mm × 3.4 mm; visual cortex −6.48 mm Bregma; and 3.0 mm × 1.2 mm. Brain visual structures were evaluated for axonal fiber tract degeneration. For all brain structures, and representative photomicrographs described below, right and left refers to the animal’s anatomy, i.e., within the lobes for the right and left hemispheres, respectively, and not the viewer’s direct perspective (i.e., handedness). We kept careful track of the orientation of the brain sections during histopathology processing, by marking the left lobe of each with a pin hole placed near the upper-left cortex.

**Figure 2:**
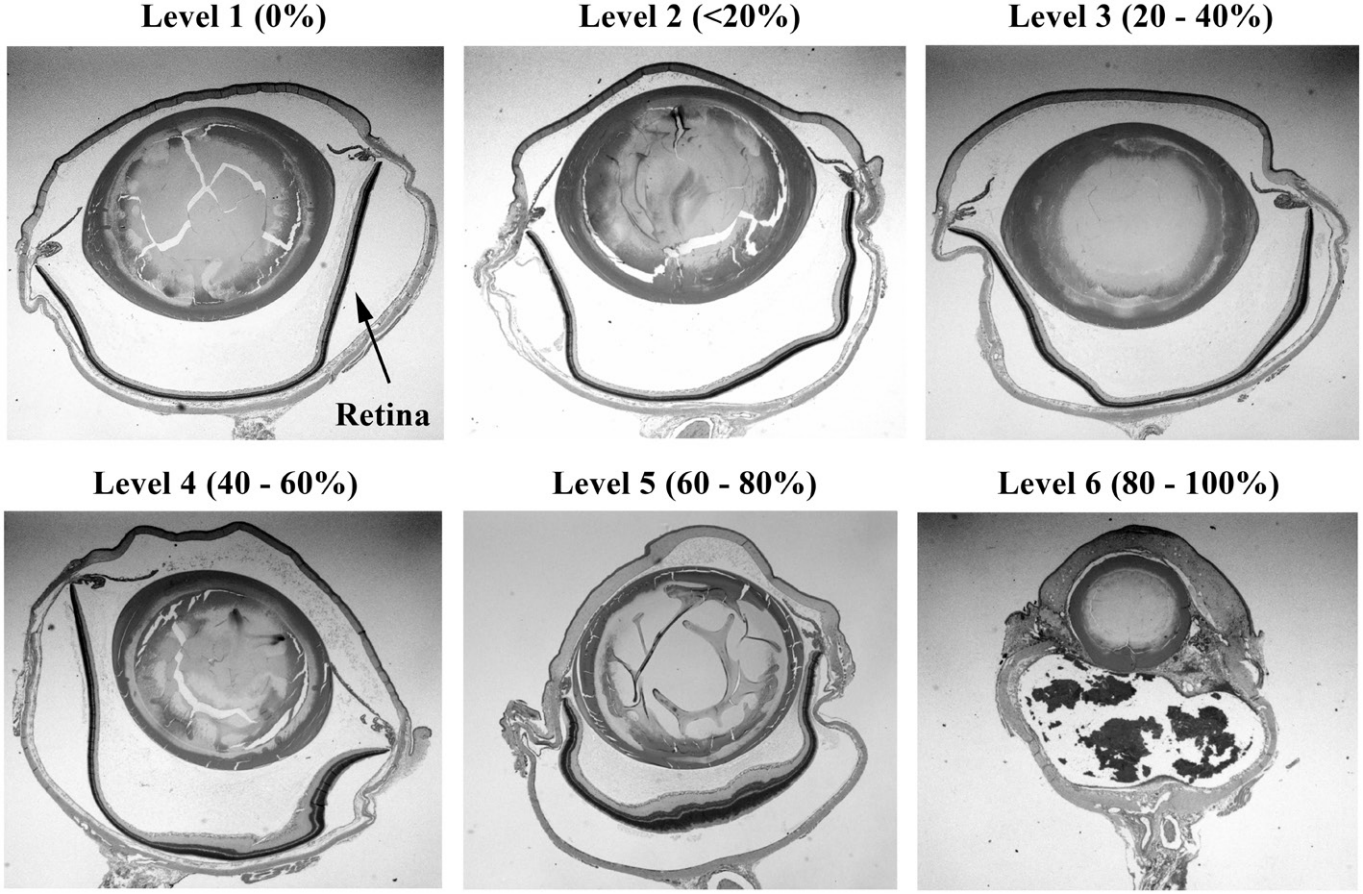
**Histopathology of eyes from right side-blasted rats at 14 days following exposure**. Representative whole eye cross sections for retina relative damage scoring scale, 1–6 (hematoxylin and eosin stained; 2× objective lens with 60% camera-zoom magnification). Arrow in first panel (level 1; none) denotes position of the retina in the eye sections. The overall extent of retinal involvement as a percentage of visible injury sites is shown above each frame.

**Figure 3:**
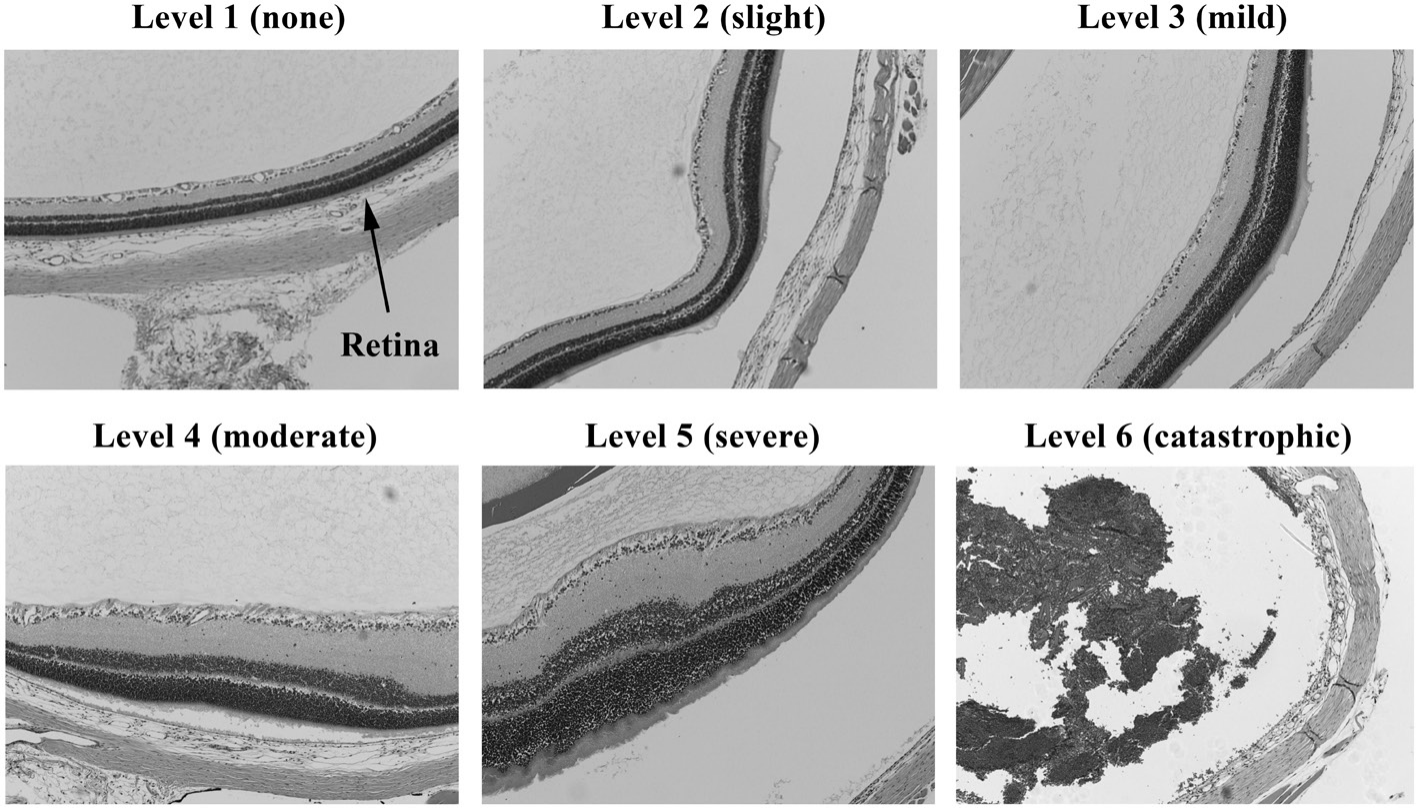
**Histopathology of eyes from right side-blasted rats at 14 days following exposure**. Enlarged views of retinas from same eyes to illustrate lesion morphologies (10× objective lens magnification). The overall degree of retina neuronal cell layer perturbations is shown above each panel. Final assignment of relative damage scores takes into consideration both the extent and degree of retinal damage present.

Injury scores for the retina, optic tract, geniculate nucleus, superior colliculus, and visual cortex were ranked on an ordinal scale by treatment blinded reviewers using values of 1–6, representing none, slight, mild, moderate, severe, and catastrophic levels of injury, respectively. Photomicrographs representing examples of scores of 1–6 for the retina are shown in Figures 2 and 3. Retinal scoring was first based on wide field examination (2× magnification) of the entire retina for plausible lesions revealed by the hematoxylin and eosin stain, which interacts with lipids and proteins to accentuate gross morphological differences. As illustrated in Figure 2, an initial score was assigned based on extent of retinal involvement at low power: 1 (0% damage), 2 (<20%), 3 (20–40%), 4 (40–60%), 5 (60–80%), and 6 (80–100%). As shown in Figure 3, lesion sites were then verified by close field examination (10× magnification) for the marked perturbations consistent with retinal degeneration (e.g., neuronal cell layer swelling, reorganization, deformation, and decomposition) with their overall width taken into account; and as warranted, the initial injury score was adjusted to better fit the overall severity of the lesions as based on these underlying morphological features seen at higher power. In our experience, the first score-assignment, as done under low power magnification, does not dramatically change with closer observations under a higher power, i.e., extent and severity closely follow each other. Through this combined scoring approach, both the extent and severity of damage were taken into account in the retinal scores. Consequently, to earn a solid score of 6, nearly all the retina had to be obliterated, as demonstrated in Figure 3, panel 6. Thus, the retina scoring is a single-scale system, where extent and severity of damage are taken into mutual consideration when mentally looking at the image; and in no manner are separate scores assigned to each that have to be physically combined later.

**Figure 4:**
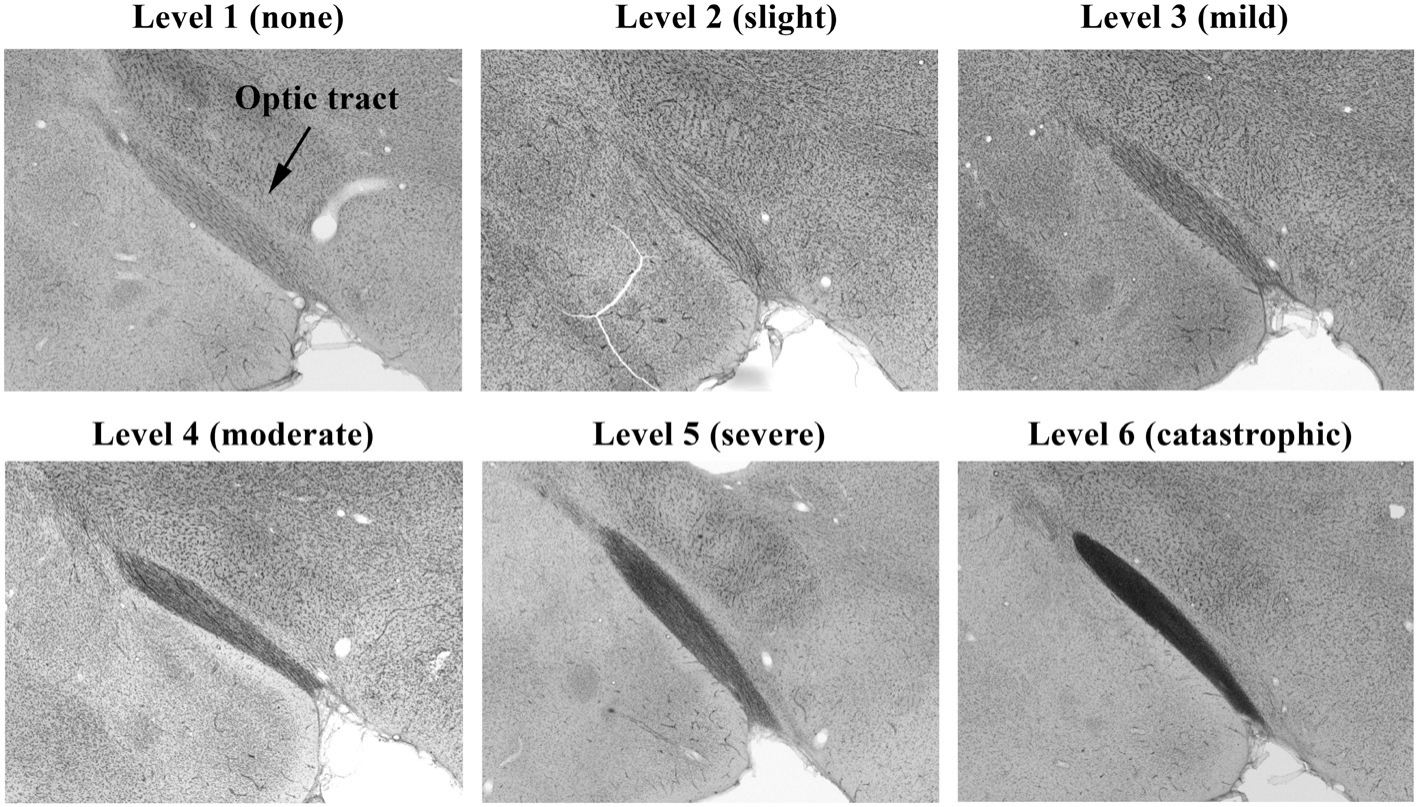
**Histopathology of brains from right side-blasted rats at 14 days following exposure**. Representative rat brain cross sections for the optic tract relative damage scoring scale, 1–6 (silver stained; 4× objective lens with 60% camera-zoom magnification). Arrow in first panel (level 1; none) denotes position of optic tract region in the brain sections. The overall degree of brain axonal damage is shown above each frame.

Photomicrographs representing examples of scores of 1–6 for the brain’s optic tract are shown in Figure 4. Brain visual center scoring was based on wide field examination (4× magnification) of the intensity of black coloration present within the entire structural region of interest (e.g., optic tract), which arose from the silver stain interacting with protein debris within degenerated axons. Exposed amino acids on these remains of axons act as favorable nucleation sites for formation of reduced silver aggregates (18). As demonstrated in Figure 4, panel 6, to earn a solid score of 6, the entire optic tract had to be densely packed with blackened axonal fibers.

### Ethical Review

All experiments and procedures were performed with the approval of the Walter Reed Army Institute of Research and the Institutional Animal Care and Use Committee.

### Statistical Methods

Electroretinogram values are on a continuous scale; thus groups of values were compared by the Student’s *t-*test. Histopathologic values are on an ordinal scale; thus groups of values were compared by Mann’s *U*-test. Many endpoints were ultimately assessed in this exploratory study. Instead of correcting *P* values for the large number of exploratory comparisons, uncorrected *P*-values are given with *P*-values ≤0.05 being stated to imply differences but not formal “statistical significance.”

## Results

### Experimental Scheme

Two experiments were performed. The first experiment was designed to determine the consequences of orientation of the rat eye relative to the blast overpressure wave, given that the rat skull is conical/stream-lined with the eyes more laterally displaced than the human skull with its more fronto-planar shape. The second phase was designed to confirm the effect of the blast wave on the eye and visual system with the animal in the previously determined optimal orientation for study. In each experiment, the amplitude of a- and b- waves on the ERG recordings for both the right eye and left eye were measured on the day prior to blast and on 1, 7, and 14 days afterward. Histopathologic findings of the right and left retina and the right and left optic tract at 2.8 mm post the optic chiasm were evaluated 14 days after the blast.

### Experiment #1

Experiment #1 compared values in animals exposed to blast whose whole face was exposed to the blast because the nose was pointed toward the compression chamber/mylar membrane in the tube (face-to-blast animals) vs. animals for whom one side of the face (the right side) faced the blast (right-to-blast animals). There were a total of 12 animals who received a face blast, 15 animals who received a right blast, and 14 sham animals who did not receive a blast but who underwent all other procedures.

#### Electroretinogram Findings

**Figure 5:**
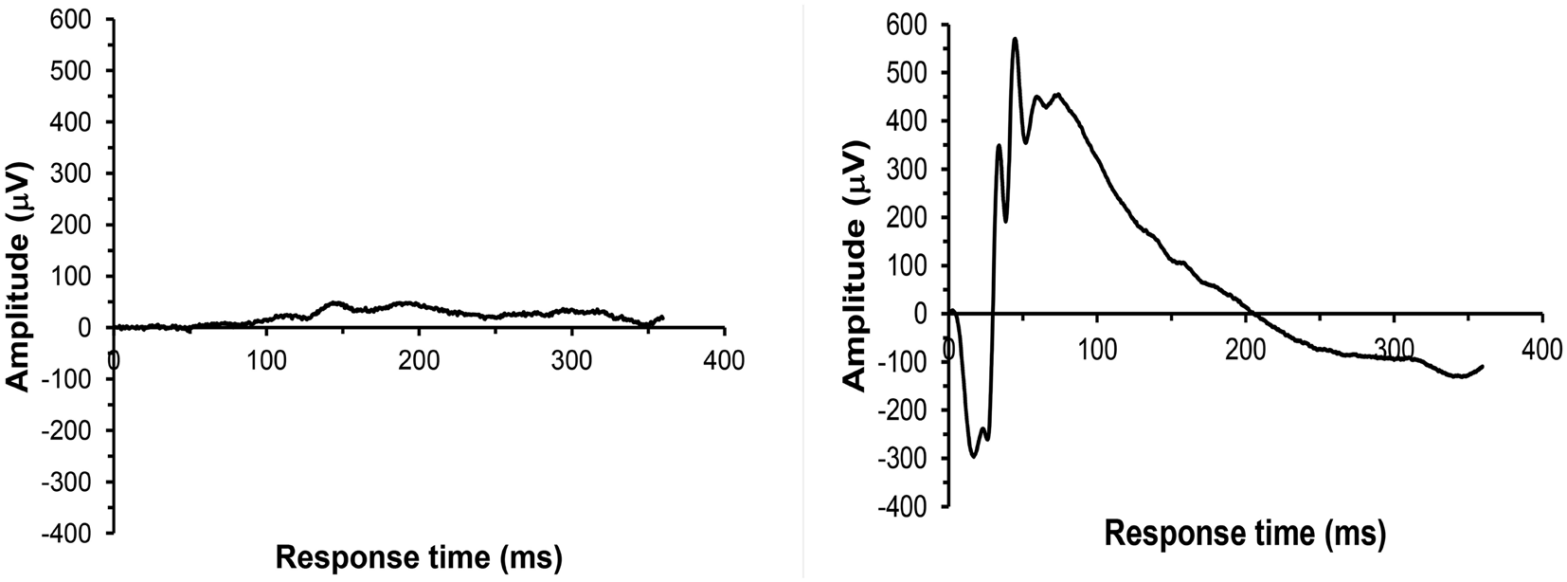
**Example of ERG recordings from a right side-blasted rat at 7 days following exposure**. Left: for the right retina, both a-wave and b-wave amplitudes are severely diminished. Right: for the left retina, the amplitudes of the initial downward deflection (a-wave) and subsequent upward deflection (the b-wave) are normal. The leading edge of the a-wave is produced by hyperpolarization of the photoreceptors, while the remainder of the wave is produced by a mixture of cells including photoreceptors, bipolar, and amacrine cells.

For all 41 animals, baseline ERG values for the right eye averaged 271 (SD = 62) μV for a-waves and 667 (SD = 154) μV for b-waves; baseline ERG values for the left eye averaged 276 (SD = 57) μV for a-waves and 682 (SD = 150) μV for b-waves. Baseline subtracted values of a-waves and b-waves at varied postinjury intervals are shown in Table 2. For face-to-blast animals, no waves at any time period showed a difference in amplitude compared to sham animals (Table 4A). For right-to-blast animals, the amplitude of a- and b-waves at 7 days post blast on the side that was blasted was diminished by 30–31% from baseline whereas the amplitude in sham animals was diminished by 4–6% from baseline (data not shown): *P* = 0.05 for change from baseline (Table 4A). An example of the diminution in the a- and-b waves in the retina facing the blast (the right retina) vs. the normal a-and-b waves in the retina away from the blast (the left retina) in the same animal is shown in Figure 5. No waves at other time periods showed a difference in amplitude in right blast animals versus sham animals.

**Table 2:** **Electroretinogram (ERG) data from experiment #1**.

Animal group	Day post blast	ERG wave	Wave side	Mean^a^	SD^a^
Sham	0 (baseline)	A	Right	282	62
			Left	286	70
	0 (baseline)	B	Right	707	161
			Left	722	194
	1	A	Right	12	65
			Left	−7	84
		B	Right	41	170
			Left	6	195
	7	A	Right	−17	69
			Left	−24	84
		B	Right	−31	157
			Left	−56	201
	14	A	Right	−40	82
			Left	−41	88
		B	Right	−92	197
			Left	−107	207
Right	0 (baseline)	A	Right	271	63
Blast			Left	279	53
	0 (baseline)	B	Right	650	149
			Left	672	137
	1	A	Right	−2	122
			Left	−7	70
		B	Right	−22	306
			Left	−17	163
	7	A	Right	−84	102
			Left	−40	67
		B	Right	−194	261
			Left	−89	177
	14	A	Right	−65	105
			Left	−37	56
		B	Right	−143	273
			Left	−67	151
Face	0 (baseline)	A	Right	254	62
Blast			Left	261	47
	0 (baseline)	B	Right	641	157
			Left	648	97
	1	A	Right	−28	106
			Left	−11	82
		B	Right	−42	247
			Left	18	194
	7	A	Right	−32	97
			Left	−17	54
		B	Right	−73	258
			Left	−26	136
	14	A	Right	−53	97
			Left	−28	47
		B	Right	−102	224
			Left	−38	137

*^a^ERG values (microvolts) are raw values for day 0 (baseline) and baseline-subtracted values for days 1, 7, and 14*.

*Negative baseline-subtracted values means that values were <baseline*.

*Number of animals: sham (14), right-blast (15), and face-blast (12)*.

#### Histopathologic Findings

Injury scores for retinas and optic tracts at 2.8 mm post the optic chiasm for the face-to-blast, right-to-blast, and sham animals on day 14 after blast are shown in Table 5A. Differences between groups are summarized in Table 6A. For face-to-blast animals, only scores for the right retina were higher (*P* = 0.02) than for sham animals. For the right-to-blast animals, injury scores for the right retina (but not the left retina), the right optic tract, and the left optic tract were markedly higher (*P* = 0.0001–0.0006) than for sham animals (Table 6A).

### Experiment #2

The purpose of experiment #2 was to corroborate the findings in right-to-blast animals of experiment #1. In experiment #2, there were 16 right-to-blast animals and 10 sham animals.

#### ERG Findings

For all 26 animals, baseline values averaged 209 (SD = 59) μV for a-waves and 518 (SD = 143) μV for b-waves; baseline ERG values for the left eye averaged 205 (SD = 49) μV for a-waves; and 502 (SD = 92) μV for b-waves. Baseline subtracted values of a-waves and b-waves at varied intervals following blast exposure are shown in Table 3. For right-to-blast animals, the amplitude of the b-waves at 7 days post blast on the side that was blasted was diminished by 24% from baseline whereas the amplitude in sham animals increased by 4% (data not shown): *P* = 0.03 for change from baseline (Table 4B). No waves at other time periods showed a difference in amplitude compared to sham animals.

**Table 3:** **Electroretinogram (ERG) data from experiment #2**.

Animal group	Day post blast	ERG wave	Wave side	Mean^a^	SD^a^
Sham	0 (baseline)	A	Right	192	51
			Left	191	46
	0 (baseline)	B	Right	474	108
			Left	477	92
	1	A	Right	35	66
			Left	26	74
		B	Right	84	159
			Left	58	159
	7	A	Right	9	65
			Left	−23	81
		B	Right	17	130
			Left	155	152
	14	A	Right	−24	65
			Left	111	81
		B	Right	−61	155
			Left	34	187
Right	0 (baseline)	A	Right	219	63
Blast			Left	213	50
	0 (baseline)	B	Right	545	158
			Left	519	90
	1	A	Right	−3	64
			Left	−16	55
		B	Right	−15	159
			Left	−39	115
	7	A	Right	−43	88
			Left	−49	73
		B	Right	−128	191
			Left	−125	135
	14	A	Right	−30	52
			Left	−47	56
		B	Right	−87	126
			Left	−93	116

*^a^ERG values (microvolts) are raw values for day 0 (baseline) and baseline-subtracted values for days 1, 7, and 14*.

*Negative baseline-subtracted values means that values were <baseline*.

*Number of animals: Sham (10) and Right-blast (16)*.

**Table 4:** **Comparison of electroretinogram data in blasted and sham animals**.

Animal group	Day post blast	ERG wave	Wave side	P^a^ vs. sham
**A. Experiment #1**				

Right	1	A	Right	0.69
Blast			Left	0.92
		B	Right	0.50
			Left	0.74
	7	A	Right	0.05
			Left	0.6
		B	Right	0.05
			Left	0.65
	14	A	Right	0.49
			Left	0.89
		B	Right	0.57
			Left	0.56
Face	1	A	Right	0.27
Blast			Left	0.90
		B	Right	0.34
			Left	0.87
	7	A	Right	0.65
			Left	0.8
		B	Right	0.62
			Left	0.65
	14	A	Right	0.71
			Left	0.63
		B	Right	0.91
			Left	0.32

**B. Experiment #2**				

Right	1	A	Right	0.17
Blast			Left	0.14
		B	Right	0.14
			Left	0.12
	7	A	Right	0.095
			Left	0.42
		B	Right	0.03
			Left	0.24
	14	A	Right	0.82
			Left	0.08
		B	Right	0.66
			Left	0.06

**C. Experiment # 1 plus #2**				

Right	1	A	Right	0.27
Blast			Left	0.34
		B	Right	0.16
			Left	0.21
	7	A	Right	0.01
			Left	0.32
		B	Right	0.005
			Left	0.26
	14	A	Right	0.54
			Left	0.3
		B	Right	0.51
			Left	0.45

*^a^*T* test*.

*ERG values are baseline subtracted*.

#### Histopathologic Findings

Injury scores for retinas and optic tracts at 2.8 mm post the optic chiasm for right-to-blast and sham animals on day 14 after blast are shown in Table 5B. Differences between groups are summarized in Table 6B. For the right-to-blast animals, injury scores for the right and left optic tracts were markedly higher (*P* = 0.0004–0.0006) than for sham animals. The right retinas of right-to-blast animals were not more injured than the right retinas of sham animals, because the latter retinas appeared to have some lesions. Since the optic tracts of the sham animals did not show injury (range of scores = 1–1.5: Table 5B), we consider that the damage to the right retinas of sham animals was due to uncontrolled-for experimental conditions (perhaps acute room light exposure in these depigmented albino rats) rather than to blast itself.

**Table 5:** **Histopathologic data**.

Animal group	Retina at 14 days post blast	Median (range)	Optic tract 2.8 mm beyond the optic chiasm at 14 days post blast	Median (range)
**A. Experiment #1**				

Sham	Right	1 (1–2)	Right	1 (1–1)
	Left	2 (1–3)	Left	1 (1–2)
Right	Right	3 (1–6)	Right	3 (1–5)
Blast	Left	2 (1–5)	Left	3 (1–6)
Face	Right	3 (1–6)	Right	1 (1–4)
Blast	Left	2 (1–5)	Left	1 (1–6)

**B. Experiment #2**				

Sham	Right	3 (1.5–3)	Right	1 (1–1)
	Left	2 (1–2.5)	Left	1 (1–1.5)
Right	Right	2 (1–4)	Right	3.25 (1–4.5)
Blast	Left	3 (1–4)	Left	3.25 (1–4)

**C. Experiment #1 plus #2**				

Sham	Right	1.5 (1–3)	Right	1 (1–2)
	Left	2 (1–3)	Left	1 (1–2)
Right	Right	2.5 (1–6)	Right	3 (1–5)
Blast	Left	2 (1–5)	Left	3 (1–6)

**Table 6:** **Comparison of histopathologic data in blasted and sham animals**.

Animal group	Retina at 14 days post blast	*P*-value vs. sham^a^	Optic tract 2.8 mm beyond the optic chiasm at 14 days post blast	*P*-value vs. sham^a^
**A. Experiment #1**				

Right	Right	0.0006	Right	<0.0001
Blast	Left	0.52	Left	0.0004
Face	Right	0.02	Right	0.64
Blast	Left	0.89	Left	0.66

**B. Experiment #2**				

Right	Right	0.78	Right	0.0004
Blast	Left	0.06	Left	0.0006

**C. Experiment #1 plus #2**				

Right	Right	0.01	Right	<0.0001
Blast	Left	0.07	Left	<0.0001

*^a^Mann–Whitney *U*-test*.

### Experiments #1 Plus #2 (Right Blast Animals)

#### ERG Findings

For right-blast animals in experiments #1 and #2 combined, the amplitude of a- and b-waves at 7 days post blast on the side that was blasted was diminished compared to the amplitude in the sham animals: *P* = 0.005–0.01 (Table 4C).

#### Histopathologic Findings

For the right-blast animals, injury scores for the right retina were higher (*P* = 0.01) than for sham animals (Table 6C). Injury scores for the right and left optic tracts at 2.8 mm post the optic chiasm were higher (*P* < 0.0001) than for sham animals (Table 6C).

### Comparison of Injury to Visual Structures Proximal to and Distal from the Blast Wave

For the first 9 animals subjected to right-to-blast in experiment #1, injury scores were assigned to succeeding structures of the visual system: retina, optic tract, dorso-lateral geniculate nucleus, superior colliculus, and visual cortex. The median (range) of injury scores was: retina −4 (1–6); optic tract −4 (2–5); dorso-lateral geniculate nucleus −3 (1–4); superior colliculus −2 (1–3.5); and occipital cortex −1 (1–1.5).

## Discussion

Blast exposure can produce significant injury to all components of the visual system. The visual system begins with the eye globe, extends via the retinal photoreceptors through the optic nerves to the optic chiasm, continues postchiasm along the brain optic tracts into the lateral geniculate/superior colliculus substructures, and terminates at the occipital cortex. We have developed a rat model to study the effects of primary blast overpressure on the retina and optic tract.

At the optic chiasm, approximately 50% of retinal ganglion cell and interconnecting optic nerve fibers cross the midline in humans whereas about 90% cross the midline in the rat (19). The position of the eyes on the head represents a trade-off between binocular vision and field of vision. Eyes placed on the front of the head have much more overlap thus binocular depth perception, which is useful for predators such as humans. Eyes placed on either side of the head gives a large field of vision, which is advantageous for prey such as rats.

We first determined whether the optimal cranial orientation for study in rats was with the face or with just the right eye directly facing the blast wave. In the face-to-blast orientation, neither the ERG nor retinal and optic tract histopathology suggested that reproducible injury had occurred. A possible explanation for the failure of the face-to-blast model is the wedge shaped architecture of the rodent’s face, such that the nose and anterior facial structures but not the eye itself receives the brunt of the blast wave in this orientation. Likely, the bulk of the wave flows aerodynamically around the head and thus past the eyes in a face-to-blast orientation.

We then reasoned that a right eye-to-blast orientation might provide greater exposure of the rat’s retina to blast. Rats exposed on one side of the face to shock-tube blast demonstrated retinal functional injury in each of the two experiments. By day 7, after a blast facing the right eye, ERG recorded a- and b-waves from the right eye were markedly diminished compared to sham (non-blasted) controls. As wave amplitude is a summation of measurements from similar neuronal cell populations, the wave patterns obtained are consistent with tissue damage. Specifically, the a-wave measures photoreceptor cells, and the b-wave is mostly produced by bipolar and amacrine cells, all populations being integral retinal constituents and necessary for vision. Examination of implicit times to peak amplitudes in right-eye blasted animals, however, did not show any significant deficits, implying that the remaining population of viable retinal neurons is firing at a normal rate (data not shown). There was no difference between a- and b-waves from the left eye, which was on the opposite side of the animal from the blast, compared to sham controls. Changes in the right-eye ERG occurred by day 7 and remitted by day 14 after the blast. Retinal histopathology on day 14 after the right-sided blast showed a difference from sham controls on the right side but not on the left side. Since histopathology was only performed on day 14 post blast, we do not know whether histopathological retinal injury is present at an earlier time point or how long the injury lasts beyond 14 days.

The ERG and histopathology derived data establish that our eye to blast-tube model is suitable to investigate the effect of primary blast on the retina. Data from this eye-to-blast model in the rat, with its conical skull, may be relevant to face-to-blast exposure of the human skull with its more planar shape.

The retinal injuries that we observed are consistent with a progressive degeneration of the constituent neuronal cell layers; especially the lower-most one comprised photoreceptor cells. These highly specialized neurons are structurally fragile and interlocked with the retinal pigmented epithelium (RPE) lining the choroid at the back of the eye; thus, they would be highly susceptible to damage by the shearing forces produced by the blast wave. Maintaining contact with the RPE is essential for proper renewal (i.e., shedding) of the photoreceptor’s outer segment extensions, as well as for the cell’s nourishment; and complete detachment of the retina leads to a highly aggressive neurodegenerative state. Presence of retinal degeneration, with an intact retinal – RPE interface, might still be amenable to treatment with drug-based therapeutics, which we ultimately want to use our blast model to investigate.

We noted in some blasted eyes that the photoreceptor cells show distorted growth of their outer segments (see Figure 3; level 5), which suggests the retinal degeneration is in part due to a separation from the RPE/choroid. Our histopathology methods, however, cannot be used to validate the presence of retinal detachments, due to possible artifact displacement of the retina during microtome sectioning of the fixed eyes (paraffin blocked). Retinal detachments (commotio retinae) from blast exposure have been observed in humans and mice, using ophthalmoscopy and optical coherence tomography to look inside the eye while *in vivo* (4, 13, 20–23). If retinal detachments are also occurring in our model, then treatment approaches would be limited to exploring surgical strategies for reattachment. Significantly reducing the blast pressure to guarantee the absence of retinal detachments, however, might lead to an undesirably lower incidence of detectable retinal injuries in the animals for properly carrying out neuroprotective drug challenge studies.

In right-side blasted rats, histopathologic damage to both right and left optic tracts at 2.8 mm post the optic chiasm was also present at day 14 post blast. In both experiments, the injury scores for left optic tracks were as elevated as scores for right optic tracts, when each anatomical region was compared to sham animals.

The mechanism by which both retinal and optic tract injury is caused by primary blast waves could be direct retinal cell layer damage followed by anterograde degeneration of brain visual pathway tract fiber bundles: degeneration from retina to optic tracts. It is known that traumatic injuries to the retina produce anterograde degeneration of axonal fibers feeding into the brain starting at the retina ganglion cell layer (5, 6). Indeed, specific destruction of the retinal ganglion cell layer following ocular blast-injury in mice has been reported, as determined by ERG response to an alternating light-pattern stimulus (pERGs) and/or histopathology (13, 20–22, 24). Although retrograde degeneration of retinal ganglion cells following a lesion of the primary visual cortex is well described (in primates) (8), the progressive diminution of injury from visual retina toward the cortex in the 9 rats assessed in experiment #1 argues against the primary lesion in our model being cortical followed by retrograde retinal injury.

An anterograde mechanism of injury propagation in our model does not, in isolation, fully explain the similar damage to the left and right optic tracts beyond the optic chiasm. Since approximately 90% of retinal fibers cross the midline at the optic chiasm in rat, for right-blast experiments, the left postchiasm optic tract would be expected to be more injured than the right postchiasm optic tract. Other mechanisms that may be contributing to right optic tract damage in our model are wave translations/reflections through the skull or through the rest of the body (see below). Perhaps, an alternative to silver stain as a detection method, such as immunohistochemistry using axonal specific proteins (e.g., neurofilament light or heavy chains), would have allowed greater discrimination of optic tract injury properties.

Although there are many reports on the broad subject of TBI on the visual system in animals, we have found a total of 15 published papers that specifically characterize simulated blast wave injuries to the visual system in animal models: 4 reports using rats and 11 reports using mice (Table 1, column 2). Of the methods done to simulate the blast wave injury, eight were by direct air-blast to the eye from a modified paint ball gun, two by a head-only air-blast (i.e., the animal was shielded below the neck) inside an open chamber (an unfocused blast), four by whole body blast waves inside a shock tube (a blast with Friedlander waveform), as well as the one use of whole body blast waves from open-field detonation of explosive charges (Table 1, column 3). Impact pressure of the insult with the eye (retina) or skull (brain) ranged 10–70 psi (i.e., low to high level blast: Table 1, column 3). Outcome measures attempted were for the retina, optic nerve, and/or brain visual centers: gross pathology, histopathology, immunohistochemistry, dendritic field analysis, electron microscopy, ERGs, single retinal cell/multi-electrode array recordings, intraocular pressures, pupil constriction response, tear production, optical coherence tomography, visual acuity (optokinetics), vision-based behavioral tests, Western blots and ELISAs of apoptotic and edema proteins, cytokine immunoassay arrays, oxidative stress marker assays, and neurotransmitter assays (Table 1, column 4). All of these studies reported deficits consistent with neuronal cell degeneration within the retina, optic nerve, and/or brain visual centers (Table 1, column 5). All five of the papers reporting retinal signaling defects by ERG along with corresponding retinal degeneration by histopathology employed mice and fired a high velocity air rifle directly at the animal’s cornea (20–22) or exposed just the head to unfocused air-blast in an open chamber (13, 24).

Our model utilizes high fidelity simulated air blast waves (Friedlander waveforms) generated by a shock tube to induce injury assessed by ERG, a fundamental retinal functional parameter, concomitantly with retinal and optic tract histopathology. The magnitude of this blast wave – approximately 20 psi for 6 ms – is comparable to the threshold level needed for one representative human biological injury, lung damage (25). We view our model as being reasonably comparable to what a soldier might experience from primary shock waves caused by explosive devices in the field (26).

Shock tube-based animal models for blast do possess some intrinsic flaws. Multiple rarefaction waves can be generated off of the walls of the tube and frame or sling of the animal holder that can strike the subject and add to the injuries. Likewise, for the “side-to-blast” orientation that we tested, visual structure injury can be partially dependent on wave translation through the skull and/or wrapping around it to strike the opposite side. Also, since a whole body blast exposure is done, there are other factors that can influence the injury severity, such as body structure (bone density) and physiological status (immediate blood pressure) of the subject. Finally, direct displacement of the eyes (retinas) in the sockets and the brain in the skull could lead to subsequent neurodegeneration from the shearing forces that could propagate in an anterograde or retrograde direction, respectively, along the axonal fibers.

Future improvements of our shock tube generated blast model may include looking at visual system injury over a wider range of conditions that might be encountered by soldiers in the field: a reasonable range of pressures (10–30 psi), repetitive blasts [multiple blasts at close (1 min) intervals], combined primary and secondary insults (for our model: a blast followed by a weight-drop induced skull-concussion). Longer times post blast should be examined to judge if the rebound of retinal signaling at 14 days post blast will be maintained or is transient and will be succeeded by a return of retinal damage (20). Optical coherence tomography, concurrent ERG and cortical evoked potentials assessments to a light stimulus, and visual acuity measurements would strengthen future visual assessment models.

Soldiers are issued protective goggles in the field, but blast-induced eye injuries will always be of great risk due to non-compliance of wear, blast wave penetration, or the goggles being blown off the face. Indeed, the incidence of closed eye injuries in blast-exposed soldiers underscores the serious retina damage and long lasting impairments in vision that often results from blasts (4, 27). Our data demonstrating ERG and histopathologic damage specific to blast-exposed retina and optic tracts suggests that this rat model may be useful to investigate therapeutic interventions, e.g., novel anti-inflammatory drugs, to counter primary blast effects on the retina.

## Author Contributions

JM, KS, TO, JB, and JL: design/acquisition/analysis/interpretation of work; draft/revise manuscript; approve manuscript; accountable for manuscript. MH: design/acquisition/analysis/interpretation of work; draft manuscript; approve manuscript; accountable for manuscript.

## Disclaimer

The contents, opinions, and assertions contained herein are private views of the authors and are not to be construed as official or reflecting the views of the Department of the Army or the Department of Defense.

## Conflict of Interest Statement

The authors declare that the research was conducted in the absence of any commercial or financial relationships that could be construed as a potential conflict of interest.
